# Neonatal Sepsis Due to Bacillus subtilis

**DOI:** 10.7759/cureus.17692

**Published:** 2021-09-03

**Authors:** Panagiotis K Lampropoulos, Despoina Gkentzi, Sotiris Tzifas, Gabriel Dimitriou

**Affiliations:** 1 Pediatrics, University Hospital Patras, Patras, GRC; 2 Pediatrics/Infectious Diseases, University Hospital Patras, Patras, GRC; 3 Pediatrics and Neonatology, University Hospital Patras, Patras Medical School, Patras, GRC

**Keywords:** neonatal sepsis, subtilis, immunodeficiency, antimicrobial resistance, bacillus

## Abstract

*Bacillus subtilis* is a gram-positive bacillus, commonly found in the environment (also as an endospore) and in the human gut (in a carrier-state), being considered as a bacterium of minimal virulence. We present a rare case of late-onset neonatal sepsis with multiple positive blood cultures and isolation of *B. subtilis* as the causative pathogen.

## Introduction

*Bacilli *are gram-positive, aerobic or facultative-anaerobic, rod-shaped bacteria [1]; in general, *Bacilli *are able of forming endospores [2], with subsequent resistance to various environmental conditions (such as heat, cold, radiation, medical disinfectants), thus gaining the potential of causing various contaminations [3]. In humans, clinically significant *Bacilli* are *B. anthracis* (the cause of anthrax) and *B. cereus* (causes food poisoning); other *Bacilli *species are only infrequently causing diseases in humans apart from the immunodeficient individuals; further on, *Bacillus spp *produce several metabolites, enzymes and antibiotics (e.g., bacitracin, polymyxin), and they are used frequently in pharmaceutics, medicine and agriculture (e.g., food preservation, use as probiotics) [4]. Only infrequently serious neonatal infections caused by *Bacillus spp* have been described in the literature [5,6]. By this means, the case of neonatal sepsis due to *Bacillus subtilis* that we describe can be considered of particular interest for paediatricians and neonatologists.

## Case presentation

A low-birth-weight (LBW) and small for gestational age (SGA) late premature neonate (birth weight 1,730g at 36th week of gestation), was born vaginally due to mild vaginal haemorrhage (≥ 24 hours) and the onset of labour in the local district hospital. Of note, the mother did not have any antenatal follow-up and she belonged to Roma ethnic group. Foul-smelling amniotic fluid and an Apgar-score of 7/9 with adequate postnatal respiratory and circulatory adaptation were reported at the local hospital. Due to prematurity and LBW, the neonate was transferred to our tertiary Neonatal Intensive Care Unit (NICU) (University Hospital of Patras, Greece) for further care where after full sepsis workup initially received empirical antibiotic treatment with intravenous ampicillin and gentamicin. On day 3 of life, cefotaxime was added due to raised infection markers, and on day 6 of life, due to infrequent desaturations, minimal abdominal distention and positive occult blood tests, feeding was stopped. A relatively stable clinical course was documented over the next three days and blood cultures were negative up to that point (which could be attributed to the only sporadical entry of pathogens in the bloodstream or insufficient blood culture samples). On day 9 of life, clinical deterioration occurred, with pallor, mottling, grunting, and apnoeas; sepsis workout, intubation and mechanical ventilation followed. On day 10 of life, a blood culture came back positive (about 12 hours after the sample collection), and on day 11, *B. subtilis* was identified as the responsible pathogen. Positive blood cultures drawn in subsequent days confirmed this finding. On day 12, the baby had raised inflammatory markers (CRP of 25 mg/dL with cut-off value 0.6 mg/dL), cerebrospinal fluid pleocytosis/meningitis (140 cells/mm^3^, negative cerebrospinal fluid cultures), and bilateral diffuse consolidations on the chest x-rays. The subsequent antibiogram showed sensitivity to meropenem (MIC 0.19), vancomycin (MIC 0.38), amoxicillin-clavulanic (MIC 0.38), ticarcillin-clavulanic (MIC 1.0), rifampicin, sulphamethoxazole-trimethoprim, fusidic, tetracycline, erythromycin, kanamycin, gentamicin, and resistance to ampicillin. Consequently, on day 14 the treatment regimen was changed to meropenem and vancomycin, and under the new treatment, the clinical condition of the neonate steadily improved. Concluding, 8 (eight) serial positive blood cultures with *B. subtilis* (over a period of seven days) were documented (identification of pathogens with VITEC 2 System, BioMérieux S.A., Marcy I´Etoile, France). It is worth mentioning that no other site cultures (cerebrospinal fluid, urine, catheter tips, etc.) were positive during the treatment. The neonate received the aforementioned treatment course up until the 28th day of life, and completed his treatment and stay in our NICU without side effects or major complications. Further on, the baby remained well in our post-discharge outpatient follow-up up until the 12th month of age.

## Discussion

*B. subtilis* is a Gram-positive, rod-shaped bacterium, usually found in soil. For a long, it has been considered as an obligative aerobe microorganism, though recently was characterized as also facultative anaerobe. *B. subtilis *has flagellates showing a decent capacity of motility in liquids, and under tough environmental settings (such as heat, cold, radiation, medical disinfectants) is also capable of forming endospores, which contributes significantly to its widespread existence in nature [7]. *B. subtilis* inhabits frequently the human gastrointestinal tract in a carrier-state [8] and is considered non-harmful to humans.

On the other hand, neonates are a vulnerable group to infection, as a result of multiple factors. Low neutrophil count, decreased neutrophil function, and insufficient complement titles lead to immature immune responses. Further on, fragile skin, immature-vulnerable gut (with bacteria overgrowth) and impaired mucus clearance increase the susceptibility to infection. Moreover, invasive medical procedures can facilitate the entry of pathogens into the bloodstream or the respiratory tract, and the overuse of antibiotics in the NICU setting drives the growth of multidrug-resistant pathogens. Consequently, neonates are considered as facultative immunocompromised hosts [9]. 

Our neonate presented a fulminant clinical course of late-onset neonatal sepsis. Unexpectedly, *B. subtilis* was initially identified as the causing pathogen. Considering its ubiquitous presence and the harmful nature of *B. subtilis* in humans, the possibility of sample contamination was also taken into account. The finding was confirmed by subsequent multiple positive blood cultures, and the appropriate according to antibiogram therapy was initiated and completed. No other culture (cerebrospinal fluid, urine, stool, catheter tips, milk preparations, surfaces, water supplies, ventilators) was found to be positive, and no other case of neonatal sepsis due to *B. subtilis* occurred at the same period. Our patient completed his stay in our NICU without major complications and presented in our follow-ups free of morbidity.

## Conclusions

*B. subtilis* is found commonly in nature and also in the human gastrointestinal tract, considered a non-harmful bacterium and flora. Our case underlines that common states of immunodeficiency in the neonatal period can lead to a serious infection due to* B. subtilis*. Consequently, neonatologists, paediatricians, microbiologists and nursery staff should always be aware of local epidemiological data, resistance patterns in a given setting (NICU), and the possibility of infection due to rare bacteria, such as *B. subtilis*.
